# Authenticity and rizz – What do they have to do with journal editors and researchers?

**DOI:** 10.4069/kjwhn.2023.12.18

**Published:** 2023-12-28

**Authors:** Sue Kim

**Affiliations:** Mo-Im Kim Nursing Research Institute, College of Nursing, Yonsei University, Seoul, Korea

This time of the year brings a sense of excitement, as various dictionaries announce the ‘Word of the Year’ that marks our preoccupations, curiosity, and reflects our ethos. Although several dictionaries announced words related to the proliferation of artificial intelligence (AI), e.g., *hallucination* [1], two major dictionaries selected divergent alternatives. Merriam-Webster selected *authenticity* [2] considering the number of lookups but also in relation to a likely “crisis of authenticity” in our era of deepfakes and post-truths [3]. On a very different tangent, Oxford Word of the Year is *rizz*, taken from the word charisma and defined as ‘style, charm, or attractiveness; the ability to attract a romantic or sexual partner’ [4]. As I looked back on the milestones of the *Korean Journal of Women Health Nursing* (KJWHN), I couldn’t help but ponder how these words might offer a timely message for journal editors and researchers alike.

## Journal accomplishments

This year KJWHN was honored and elated to be indexed in MEDLINE [5], a major feat for a regional journal in the field of nursing, as only approximately 11% of journals that apply to MEDLINE are accepted [6]. This is evermore significant as we are also one of a small cadre of nursing journals simultaneously indexed in PubMed Central (PMC) [5]. Another milestone was publishing a special issue, which was a first for KJWHN. The September 2023 issue focused on “Digital era education for women’s health and wellbeing” (https://kjwhn.org/current/index.php?vol=29&no=3) and covered expert opinion on opportunities and challenges for AI-integrated healthcare and healthcare education [7], as well as reviews and original research on quality evaluation, digital literacy, and virtual reality use in the classroom.

## Journal metrics

Table 1 presents data on manuscripts submitted to KJWHN as of December 10, 2023. The increase in unsolicited manuscripts, from 63 in 2022 [8] to 80 in 2023, suggests a boost from being indexed in PMC and the Emerging Sources Citation Index (ESCI) in 2022. A notable increase in international submissions was also seen. However, compared to seven unsuitable manuscripts in 2022 [8], a substantial increase of 30 manuscripts was also observable this year. These editorial or ‘desk’ rejections were largely due to incongruency with our aims and scope or concerns with high percentages in screening for plagiarism. Related to these increases in numbers, however, the editorial board has experienced challenges with limitations in resources and time, and subsequent fatigue in editors and reviewers. This is likely to have affected the time from submission to acceptance, which was roughly 56 days in 2022 [8] but lagged slightly to 85 days in 2023.

## Applying the Words of the Year to scholarly publishing

Considering our accomplishments and journal metrics, you could say KJWHN has proven it has rizz enough, evidenced by becoming indexed in MEDLINE and attracting an increase in submissions, especially from overseas. However, our challenge is to stay true to our identity and mission, i.e., striving for an authentic presence as a scholarly journal committed to women’s health nursing.

In this line, a continuous challenge is to communicate more effectively with potential authors to recognize the journal’s aims and scope as well as our emphasis on international standards for scholarly work, e.g., advocating the use of reporting guidelines, clinical trial registration for human intervention studies, data sharing statements, etc. [8]. Another real challenge is to widen the pool of reviewers to facilitate the review process. A strategic plan would include preparing junior researchers as reviewers, showing respect and appreciation for their participation, while monitoring whether reviewers might show signs of reviewer fatigue. I take this opportunity to welcome inquiries about becoming a reviewer or how to improve reviewing skills.

Researchers could also apply these two Word of the Year terms to scholarly publications. Firstly, authenticity is central when writing the manuscript for dissemination. Merriam-Webster defines authentic as “not false or imitation; true to one’s own personality, spirit, or character” [9]. Thus, authentic manuscript writing can be interpreted as being true to the spirit and main message of the study findings. It may be tempting to think research rizz involves verbose writing or strictly following a ‘template-style’ flow of writing, as is often seen in early-stage researchers. It is worthwhile, however, to remember that wordy manuscripts do not necessarily showcase productivity or value; they rather run the risk of being redundant and cliché, and may subsequently end in vague, superficial implications. In other words, be true to your study’s main message and aim for succinctness and clarity. An example is to bring the main research aim and dependent variable to the forefront of the Discussion section, focusing on interpretation and implications, rather than flooding the text with what was already presented in the Results section.

## Appreciation for 2023 reviewers

I wish to acknowledge the following dedicated reviewers who have supported KJWHN this year:

Ahn, Suk Hee (Chungnam National University)

Bae, Kyungeui (Dongseo University)

Chae, Hyun Ju (Joongbu University)

Cheon, Suk Hee (Sangji University)

Cho, Insook (Inha University)

Cho, Ok-Hee (Kongju National University)

Choi, Hyunkyung (Kyungpook National University)

Choi, Mi Jin (Chodang University)

Choi, So Young (Gyeongsang National University)

Chung, Chae Weon (Seoul National University)

Chung, Mi Young (SunMoon University)

Ha, Ju Young (Pusan National University)

Han, Jeehee (Chung-Ang University)

Haruna, Megumi (University of Tokyo)

Hong, Sehoon (Cha University)

Huh, Sun (Hallym University)

Hwang, Kyung Hye (Suwon Science College)

Jang, Hyun-Jung (Catholic Kkottongnae University)

Jeong, Geum Hee (Hallym University)

Jo, Myung Ju (The Catholic University of Korea)

Jun, Eun-Young (Daejeon University)

Kang, Saemi (Gyeongsang National University)

Kang, Sookjung (Ewha Womans University)

Kim, Haewon (Seoul National University)

Kim, Hee Kyung (Kongju National University)

Kim, Hye Young (Keimyung University)

Kim, Hyun Kyoung (Kongju National University)

Kim, Jeung-Im (Soonchunhyang University)

Kim, Joungyoun (University of Seoul)

Kim, Kwang Ok (Dongju College)

Kim, Kyungwon (Daegu Haany University)

Kim, Miok (Dankook University)

Kim, Mi Jong (Hannam University)

Kim, Mi Young (Woosuk University)

Kim, Moonjeong (Pukyong National University)

Kim, Myoung hee (Semyung University)

Kim, Su Hyun (Nambu University)

Kim, Sun-Hee (Daegu Catholic University)

Kim, Sun Ho (Chungbuk National University)

Kim, Yoonjung (Konyang University)

Kim, Young Man (Jeonbuk National University)

Kim, YoungJu (Daejeon Health Institute of Technology)

Kim, Yun Mi (Eulji University)

Ko, Eun (Sunchon National University)

Lee, Ju-Young (The Catholic University of Korea)

Lee, Kyoung-Eun (Texas A & M University)

Lee, Sun-Kyoung (Seoul Womens College of Nursing)

Lee, SunHee (Gimcheon University)

Nho, Ju-Hee (Jeonbuk National University)

Park, Seo A (Gyeongbuk College of Health)

Park, So Mi (Yonsei University Wonju)

Seo, Minjeong (Gyeongsang National University)

Shimpuku, Yoko (University of Hiroshima)

Song, Ju-Eun (Ajou University)

Song, Young A (Ansan University)

Shin, Gi Soo (Chung-Ang University)

Sung, Mi Hae (Inje University)

Yeom, Gyejeong (JEI University)

Yoo, Hana (Daejeon University)

Yoon, Ji Won (Shinhan University)

For the “Reviewer of the Year 2023,” the journal congratulates the following reviewers:

• Jeung-Im Kim (Soonchunhyang University)
• Joungyoun Kim (University of Seoul)
• Minjeong Seo (Gyeongsang National University)

And special recognition goes to our “Editor’s Pick 2021–2022,” as the most cited manuscripts in the Scopus database for the previous 2 years period.

• Lee EH. [Psychometric properties of an instrument 2: structural validity, internal consistency, and cross-cultural validity/measurement invariance]. Korean J Women Health Nurs. 2021 Jun 30;27(2):69-74. Korean. https://www.doi.org/10.4069/kjwhn.2021.05.18.
• Cho KA. Korea’s low birth rate issue and policy directions. Korean J Women Health Nurs. 2021 Mar 31;27(1):6-9. https://www.doi.org/10.4069/kjwhn.2021.02.16.

As we look towards the future, the journal will continue to keep true to our identity and scope, committing to quality, trustworthiness, and communication with potential authors. Please join us in making an impact on women’s health by becoming a reviewer and/or considering the journal for submission of quality studies on women’s health nursing.

## Figures and Tables

**Table 1.** Basic statistics on manuscripts submitted to the *Korean Journal of Women Health Nursing* in 2023

Category	Data	Notes
Commissioned manuscripts (n)	10	5 Editorials, 4 Issues & Perspectives, and 1 Invited paper
Unsolicited manuscripts (n)	80	
Accepted manuscripts (n)	30	
Non-accepted manuscripts (n)	46	15 Rejected, 30 rejected before review (unsuitable), 1 withdrawn
Manuscripts reviewed and determined (n)	45	30 Accepted, 15 rejected
Manuscripts under review or revision (n)	14	
Acceptance rate (%)	50.8	30 of 59
Average time from submission to acceptance (day)	84.9	
